# LncRNA KCNQ1OT1 Secreted by Tumor Cell-Derived Exosomes Mediates Immune Escape in Colorectal Cancer by Regulating PD-L1 Ubiquitination *via* MiR-30a-5p/USP22

**DOI:** 10.3389/fcell.2021.653808

**Published:** 2021-07-19

**Authors:** Di Xian, Liangbo Niu, Jie Zeng, Lei Wang

**Affiliations:** ^1^Department of Emergency Surgery, Sichuan Provincial People’s Hospital, University of Electronic Science and Technology of China, Chinese Academy of Sciences Sichuan Translational Medicine Research Hospital, Chengdu, China; ^2^Department of Emergency Medicine, Sichuan Provincial People’s Hospital, University of Electronic Science and Technology of China, Chinese Academy of Sciences Sichuan Translational Medicine Research Hospital, Chengdu, China

**Keywords:** colorectal cancer, lncRNA KCNQ1OT1, PD-L1, immune escape, exosomes

## Abstract

**Background:** This study tried to explore the mechanism of long non-coding RNA (lncRNA) KCNQ1OT1 in tumor immune escape.

**Methods:** Gene Expression Omnibus (GEO) and microarray analysis were used to screen the differentially expressed lncRNA and microRNA (miRNA) in normal tissues and tumor tissues. Quantitative reverse transcription PCR (RT-qPCR) was used to quantify KCNQ1OT1, miR-30a-5p, ubiquitin-specific peptidase 22 (USP22), and programmed death-ligand 1 (PD-L1). The interactive relationship between KCNQ1OT1 and miR-30a-5p was verified using dual-luciferase reporter gene assay and ribonucleoprotein immunoprecipitation (RIP) assay. Cell Counting Kit (CCK)-8, clone formation, wound healing, and apoptosis are used to detect the occurrence of tumor cells after different treatments. Protein half-life and ubiquitination detection are used to study the influence of USP22 on PD-L1 ubiquitination. BALB/c mice and BALB/c nude mice are used to detect the effects of different treatments on tumor growth and immune escape *in vivo*.

**Results:** The expression of lncRNA KCNQ1OT1 in tumor tissues and tumor cell-derived exosomes was significantly increased. The tumor-promoting effect of lncRNA KCNQ1OT1 was through the autocrine effect of tumor cell-derived exosomes, which mediates the miR-30a-5p/USP22 pathway to regulate the ubiquitination of PD-L1 and inhibits CD8+ T-cell response, thereby promoting colorectal cancer development.

**Conclusion:** Tumor cell-derived exosomes’ KCNQ1OT1 could regulate PD-L1 ubiquitination through miR-30a-5p/USP22 to promote colorectal cancer immune escape.

## Introduction

Colorectal cancer (CRC) is one of the most common malignant tumors in the world. In 2012, the number of global incidences exceeded 1.3 million with nearly 700,000 deaths (Chen X. et al., 2020). Some studies predict that by 2030, the burden of CRC is expected to increase by 60%, including 2.2 million new cases and 1.1 million CRC-related deaths (Arnold et al., 2017). In recent years, the number of patients with CRC in China has also increased year by year. Tumor metastasis is one of the main problems affecting the clinical treatment of CRC. According to statistics, more than half of CRC patients will develop distant metastasis, and 80% of them have unresectable liver metastases at the time of diagnosis (Bruni et al., 2020; Xu et al., 2020). At present, studies have shown that immunotherapy may provide an ideal treatment for patients with metastatic colon cancer (Ciorba, 2013; Singh et al., 2015; Chen and Mellman, 2017; Kalyan et al., 2018). In addition, recent studies have shown that the intrinsic functional expression of programmed death receptor 1 (PD-1) contributes to tumor immune resistance. In melanoma cells, PD-1 can be activated by the ligand programmed death-ligand 1 (PD-L1) expressed by tumor cells, regulate downstream mammalian rapamycin signaling targets, and promote tumor growth, regardless of adaptive immunity. According to reports, this PD-1 signal transduction has been found to promote cancer in liver cancer cells, bladder cancer, and non-small-cell lung cancer cells (Kleffel et al., 2015; Li et al., 2017; Yao et al., 2018). Although there have been some applications of immunotherapy, the regulatory mechanism of PD-L1 in CRC itself and its role still need to be explored in depth.

Long non-coding RNA (lncRNA) is a type of RNA molecule longer than 200 nt, which does not encode protein itself (Lin and Yang, 2018). More and more pieces of evidence show that lncRNAs are important regulators of gene expression and participate in various physiological and pathological processes, including tumor occurrence and development (Xu et al., 2019). Previously, there have been studies including our research group on the function of lncRNA in CRC (Quinn and Chang, 2016; Xian and Zhao, 2019). But its role in CRC is still worthy of attention.

Exosome is a kind of extracellular vesicle with a diameter of about 40–160 nm (about 100 nm on average) that acts on communication between cells. Its biological research has been significantly developed in the past decade (Kalluri and LeBleu, 2020). Existing analytical techniques have made it possible to isolate exosomes and detect their contents, which can carry a large number of molecules that produce biological effects, including growth factors, metabolic enzymes, lncRNA, and transcription factors (Shang et al., 2020). Since the separation of exosomes from different biological samples is relatively simple, these particles can regulate key biological functions and may serve as participants in disease pathogenesis or as disease biomarkers and drug carriers (Nabariya et al., 2020). Because exosomes have the potential as a unique biological effect molecule release system, from a therapeutic point of view, exosomes are gaining in-depth research interest.

Deubiquitinating enzymes (DUBs) are members of the protease superfamily that mediate the removal of ubiquitin (Ub) from Ub-binding substrate proteins. At present, there are approximately 90 DUBs known to include USPs, OTUs, UCHs, and other families (Komander et al., 2009; Liu et al., 2020). Ubiquitin-specific peptidase 22 (USP22) belongs to the subfamily, the ubiquitin-specific processing proteases (USPs). USP22 is considered to be an oncogene because it is overexpressed in malignant tumors in several tissues. Therefore, it can be used as a biomarker to predict the recurrence and metastasis of malignant tumors (Huang et al., 2019). USP22 stabilizes these substrate proteins and inhibits their proteasomal degradation (Schrecengost et al., 2014). It is worth noting that a recent study showed that USP22 can regulate the ubiquitination of PD-L1 in hepatocellular carcinoma (HCC) cells (Huang et al., 2020). Therefore, its role in CRC has attracted the attention of this research.

We previously reported the research status of lncRNA KCNQ1OT1 in the resistance mechanism of CRC. This time we screened and explored the role of KCNQ1OT1 in inducing immune escape in CRC through bioinformatic methods and molecular biology methods. By exploring its source and way of action, it provides a preliminary basis for the future application of immunotherapy or targeted therapy in CRC.

## Materials and Methods

### Clinic Samples and Cell Culture

Twenty CRC patients receiving surgery during the period between 2014 and 2017 in Sichuan Provincial People’s Hospital, University of Electronic Science and Technology of China, enrolled in this study and offered the CRC tissue as well as adjacent normal tissues. All cases were confirmed by a pathologist, and none of them had received chemotherapy or radiotherapy before surgery. Informed consents had been written by all the patients, and the whole procedure were supervised and approved by the ethics committee of Sichuan Provincial People’s Hospital, University of Electronic Science and Technology of China. The patient information was shown in Supplementary Table 1.

Human normal colonic mucosa cell line FHC, human embryo kidney cell line HEK-293T, and human rectal adenocarcinoma cell lines SW480, SW1463, and HT-29 cells were obtained from American Type Culture Collection (ATCC). In addition, the mouse CRC cell line CT26 was also obtained from ATCC. Dulbecco’s Modified Eagle’s Medium (DMEM, Gibco, United States) that includes 10% fetal bovine serum (FBS, Gibco) was purchased for HEK-293T cell cultivation; RPMI-1640 medium (Gibco) with 10% FBS was used for other cell lines. All the cells were incubated at the condition of 37°C, 5% CO_2_.

### Microarray Analysis

RNA sequencing (RNA-Seq) data come from eight patients in this study. All sequencing data come from the Gene Expression Omnibus (GEO) database (Platforms: GPL21290 Illumina HiSeq 3000, GSE104836) (Li M. et al., 2018). The microarray analysis of miRNA differential expression comes from the animal model. All sequencing and analysis technologies come from AKSomics Biological (China). We analyzed the expression profiles of 2,219 lncRNAs using eight pairs of CRC tissues and matched adjacent tissues. The differential analysis was conducted with R 3.4.1^1^. *p* < 0.05 and log2(fold change) > 2 were set as the screening conditions. Among all the differentially expressed lncRNAs, we selected the 20 most significantly expressed and plotted heat maps for display. The patient information was shown in Supplementary Table 2.

### Animal and Tumor Models

Fifty BALB/c mice and BALB/c nude mice aged 3–5 weeks were purchased from Charles River Laboratories (China). All animals are kept in a constant temperature, constant humidity, and no specific pathogen level animal center. They had unlimited water and diet access. All animal studies were approved by the Animal Ethical and Welfare Committee of Sichuan Provincial People’s Hospital, University of Electronic Science and Technology of China. The BALB/c mice were randomly divided into four groups (NC group, OE-KCNQ1OT1 group, OE-KCNQ1OT1+miR-30a-5p mimics group, and OE-KCNQ1OT1+sh-USP22 group, *n* = 6) and subcutaneously inoculated in the right flank with 4 × 10^5^ CT26 cells (Lou et al., 2019). And the BALB/c nude mice were randomly divide into three groups (NC group, exosome (Exo) group, and Exo+sh-KCNQ1OT1 group, *n* = 6) and subcutaneously inoculated in the right flank with 1 × 10^6^ SW1463 cells. Tumor volume was calculated using the following formula: π/6 × length × width^2^.

### Cell Transfection

CRC cells for *in vitro* assays were transfected with NC, Exo, Exo+si-NC, Exo+si-KCNQ1OT1, OE-KCNQ1OT1, OE-KCNQ1OT1+miR-30a-5p mimics, and OE-KCNQ1OT1+si-USP22. All interference vectors in this experiment were purchased from GeneChem (Shanghai, China). All transfections were performed using Lipofectamine 2000 (purchased from Sigma). At 48 h after transfection, cells were harvested for transfection efficiency test. In the *in vivo* assays, CRC cells were injected into animals after being treated in the same way. All detailed procedures followed the manufacturers’ instructions.

### Isolation and Identification of Exosomes

When the FHC and SW1463 reached 80% confluence, the medium was replaced with exosome-free medium, and the supernatant was collected 48 h later. Subsequently, differential centrifugation and filtration were performed to separate exosomes from the supernatant of cells. The cell supernatant was centrifuged at 2,000 g for 20 min, then at 10,000 g for 40 min, and then filtered through a 0.22-μm sterile filter. The supernatant was then ultracentrifuged at 100,000 g for 70 min, resuspended in phosphate buffered saline (PBS), centrifuged at 100,000 g for 70 min to remove any remaining RNA, and diluted with a mixture containing PBS and RNase I.

The morphology of exosomes was observed under transmission electron microscope (TEM). Images were obtained with HT7700 TEM (Hitachi, Japan) at 120 kV. The particle size and concentration of exosomes were determined by nanoparticle tracking analysis (NTA). Western blotting was used to detect exosomal specific markers.

### Cell Proliferation Ability Test

For Cell Counting Kit (CCK)-8 assay, we employed Cell Counting Kit (Yeasen, China) to determine the viability of cells. The optical absorbance was measured at 450 nm. All operation steps are carried out in accordance with the instructions in the kit manual.

### Transwell Assay and Flow Cytometry Analysis

To examine cell invasion, Matrigel (BD Biosciences, United States) diluted with serum-free medium was added to the upper chamber of the Transwell plate, which was then incubated at 37°C overnight. For cell migration tests, Matrigel was not added. The cell suspension (1 × 10^5^) was transferred into the upper chamber, and the lower chamber was filled with 500 μl DMEM containing 10% FBS. Following incubation for 24 h, culture medium containing migratory or invasive cells in the lower chamber was removed, and cells were detected under an inverted optical microscope (Nikon, Japan).

In terms of flow cytometry analysis, the number of apoptotic cells was counted using an Alexa Fluor^®^ 488 Annexin V/PI Apoptosis Kit (Invitrogen, CA, United States) according to the manufacturer’s protocol. The results are shown as the percentage of apoptotic cells relative to the total number of cells. All the experiments were repeated three times.

### Colony Formation Assay

Here, 1,000 cells were plated into each well of a six-well plate and were maintained in media containing 10% FBS to allow colony formation for 2 weeks, with the medium being replaced every 4 days. Later, colonies were fixed with methanol and stained with 0.1% crystal violet (Bio Basic Inc., Markham, Canada) for 15 min. The stained colonies were counted.

### Dual-Luciferase Reporter Assay

To identify the target of miR-30a-5p, putative target genes were searched using Starbase database^2^ (Li et al., 2014). Wild-type or mutated USP22 and KCNQ1OT1 3′ UTR including the predicted miR-30a-5p binding site were PCR-amplified and inserted into the pMIR-reporter plasmid. The luciferase reporter plasmids of wild-type or mutated USP22 and KCNQ1OT1 were transfected into cells, respectively, and miR-30a-5p mimics were also transfected. Luminescence was measured 48 h after transfection using a dual-luciferase detection kit (Promega, Madison, WI), according to the manufacturer’s instructions. Measurements of luminescence were performed on a GloMax 20/20 Luminometer (Promega Corporation).

### Ribonucleoprotein Immunoprecipitation Assay

Anti-AGO2 ribonucleoprotein immunoprecipitation (RIP) was performed in HEK-293T cells transfected with miR-30a-5p mimics or miR-NC. Briefly, HEK-293T cell lysates were pre-blocked with Protein G beads (Invitrogen) and then incubated with anti-AGO G beads (Pierce Biotechnology, Waltham, MA) at 4°C for 90 min. Beads were collected by centrifugation at 600 × g for 1 min, washed five times with radioimmunoprecipitation assay (RIPA) buffer and resuspended in Tris-HCl 50 mmol/L (pH 7.0). The beads were then incubated for 45 min at 70°C to reverse the crosslinks, and the RNAs that co-immunoprecipitation (IP) with anti-AGO antibodies were extracted using TRIzol (Invitrogen) following the manufacturer’s instructions and then quantified by RT-PCR.

### Flow Cytometry and Immunofluorescence

Processing the tumor tissue into a single-cell suspension referred to a previous method (Hartley et al., 2017). Cells were counted before staining with CD8APC/Cy7 and CD4-PE/Cy5 antibodies. All antibodies were purchased from BioLegend (San Diego, United States). The stained samples were analyzed on a FACS Canto II cytometer (BD Biosciences, San Jose, United States).

For immunofluorescence, a 5-μm-thick colon tumor section was blocked with 10% goat serum at room temperature for 30 min and then incubated with USP22 (Abcam) and PD-L1 (Abcam) primary antibodies at 4°C overnight. Donkey anti-goat Alexa 488 and donkey anti-rat Alexa 594 were used as secondary antibodies at room temperature for 1 h. The nucleus was stained with 4′,6-diamidino-2-phenylindole (DAPI). The image was observed through a fluorescence microscope.

### T Cell-Mediated SW1463 Cell Killing Assay

CCK-8 test, clone formation test, and apoptosis test referred to previous methods (Lim et al., 2016). In short, T cells are preactivated with CD3 antibody (100 ng/ml) and IL-2 (10 ng/ml). Then, SW1463 cells and T cells were cocultured for 24 h and then used for related treatment. CCK-8 solution (10 μl) was added to each well and incubated for 1 h. The cell viability was analyzed by a microplate reader at 450 nm. To perform the clonogenic assay, SW1463 tumor cells and activated T cells were cocultured in six-well plates. On the second day, the cells were treated and grown at 37°C for 7 days, and the surviving SW1463 tumor cells were stained with crystal violet. Colonies (50 or more cells) were counted using an inverted microscope.

### Programmed Death-Ligand 1 Deubiquitination Assay

For *in vitro* deubiquitination, HA-PD-L1 was ubiquitinated *in vitro* and separated from free ATP by centrifugation. Ubiquitinated PD-L1 (Ub-PD-L1) was then incubated with recombinant FlagUSP22 in 30 μl deubiquitination buffer containing Tris-HCl (50 mM, pH 7.4), NaCl (150 mM), dithiothreitol (DTT; 10 mM), and MgCl_2_ (5 mM). The reaction was carried out at 37°C for 2 h and then stopped by adding 25 μl of 2 × sodium dodecyl sulfate (SDS; final concentration 1%) sample buffer and boiled for 5 min. After immunoprecipitation with anti-HA antibody, PD-L1 was analyzed by Western blot with anti-ubiquitin antibody.

### Quantitative Reverse Transcription PCR

Total RNA was extracted by TRIzol (Ambion, United States). All quantitative reverse transcription PCR (RT-qPCR) processes were accomplished using the SYBR Green qPCR Master Mix (MedChem Express, NJ, United States). Amplification reactions were carried out in 7900HT Fast Real-Time PCR (Thermo Fisher Scientific, MA, United States), and results were calculated by the 2^–ΔΔCt^ method. Glyceraldehyde 3-phosphate dehydrogenase (GAPDH) and U6 were used for normalization as a control. Primers were shown in Supplementary Table 3.

### H&E Staining and Immunohistochemistry

The 5-μm tissue sections were deparaffinized in xylenol for 20 min, rehydrated with reduced ethanol concentrations (100, 90, and 70%) for 5 min, and then washed with water. For H&E staining, the slides were stained with Mayer’s hematoxylin solution for 5 min and then counterstained with eosin for 5–10 min. For immunohistochemistry (IHC), the slices were boiled in 10 mM citrate buffer for 15 min and then incubated with 5% H_2_O_2_ in PBS. After blocking with 10% FBS in PBS, the sections were incubated with primary antibodies diluted in 5% FBS in 5% PBS at 4°C overnight. Biotinylated secondary antibodies (1:200; abcam) and ExtrAvidin-peroxidase (1:1,000; abcam) diluted in PBS were added for 1 h each. Here, 3,3′-diaminobenzidine-tetrahydrochloride was used for dyeing, and hematoxylin was used for counterstaining. The slides were dehydrated, incubated in xylene, and fixed. The maximum intensity of 4–6 stained images per mouse was divided by the minimum intensity.

### Western Blot

First, proteins were isolated from extraction reagent (Thermo Fisher Scientific), then some proteins were separated and transferred to Immobilon^TM^-P membranes (Merck Millipore, Billerica, United States). Second, PD-L1, USP22, CD63, CD9, Aliex, calnexin, E-cadherin, N-cadherin, vimentin, and tubulin (Abcam) primary antibodies against mouse in the membrane were incubated at 4°C overnight. Immediately washed with TBST after incubation, membranes were hybridized with horseradish peroxidase (HRP)-linked antibody goat anti-rabbit IgG (1:2,000, Abcam) for 1 h. Lastly, antibody binding was checked by an enhanced chemiluminescence kit.

### Statistical Analysis

GraphPad Prism 6.0 software (GraphPad Software, United States) was used for statistical analysis, and data were expressed as mean ± standard deviation. Data were compared by unpaired *t*-test (differences between two groups) or one-way ANOVA (differences among groups). ^∗^*p* < 0.05 was regarded statistically significant.

## Results

### LncRNA KCNQ1OT1 Was Upregulated in Colorectal Cancer

**FIGURE 1:**
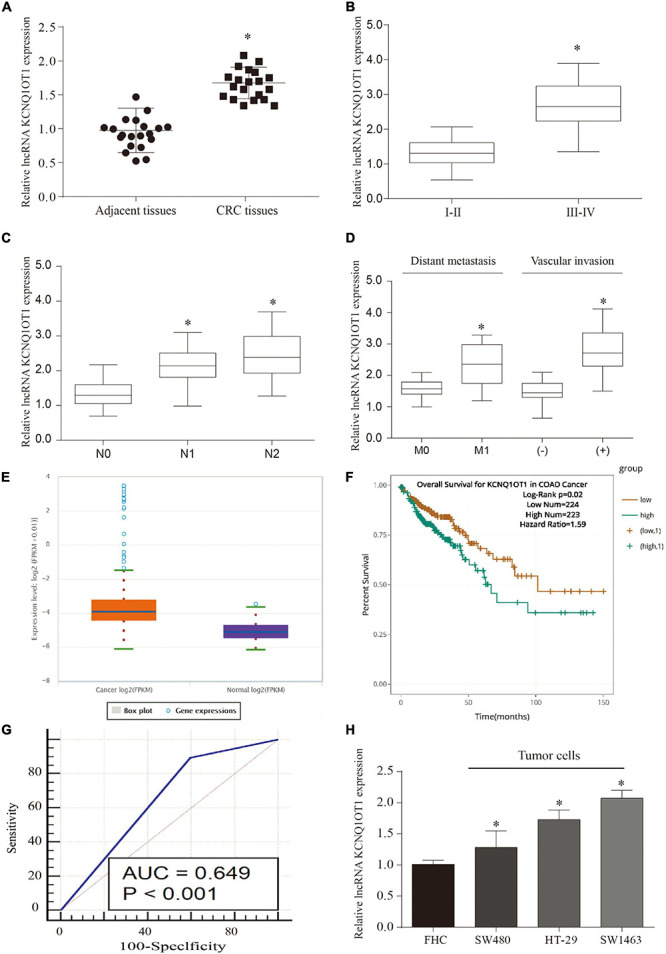
Long non-coding RNA (LncRNA) KCNQ1OT1 was upregulated in colorectal cancer (CRC). **(A)** The expression of lncRNA KCNQ1OT1 in 20 pairs of patient samples. **(B–D)** The expression level of KCNQ1OT1 was positively correlated with tumor stage, lymph node metastasis, and vascular invasion. **(E)** The expression level of KCNQ1OT1 in The Cancer Genome Atlas (TCGA) database. **(F)** The 150-month prognosis analysis of KCNQ1OT1 in TCGA database. **(G)** The receiver operating characteristic (ROC) curve was used to analyze and verify the value of KCNQ1OT1 as a clinical diagnostic index. **(H)** The differential expression of KCNQ1OT1 in colon cells and colon cancer cells. ^∗^*p* < 0.05 compared with the relative control group.

We screened eight pairs of patients with different lncRNAs in tumor tissues and adjacent tissues by GEO analysis and found that KCNQ1OT1 was significantly higher in tumor tissues than that in adjacent tissues (Supplementary Figure 1A). Our previous studies also found that it functions in drug-resistant CRC cell lines (Xian and Zhao, 2019). Therefore, we chose KCNQ1OT1 for further research. In addition, we verified that KCNQ1OT1 was significantly increased in tumor tissues compared with that in adjacent tissues (*p* < 0.05) (Figure 1A). The analysis results showed that KCNQ1OT1 expression level was positively associated with advanced stage, lymph node metastasis, distant metastasis, or vascular invasion (*p* < 0.05) (Figures 1B–D). Especially, we verified the expression of KCNQIOT1 in the CRC data in The Cancer Genome Atlas (TCGA) through Starbase data (see text footnote 2). The results showed that KCNQ1OT1 was upregulated in the cancer tissues, and its high expression is associated with poor prognostic outcome (Figures 1E,F). Furthermore, we tried to analyze its application value in diagnosis through receiver operating characteristic (ROC) curve [area under the ROC curve (AUC) = 0.649, *p* < 0.05] (Figure 1G). Finally, we used four CRC cell lines to verify the expression of KCNQ1OT1 and found that it was elevated in all CRC cell lines. But the performance is most obvious in SW1463, so we chose SW1463 as the cell line for further study (*p* < 0.05) (Figure 1H).

### Colorectal Cancer Cells Can Release a Large Amount of Exosomes Containing KCNQ1OT1

**FIGURE 2:**
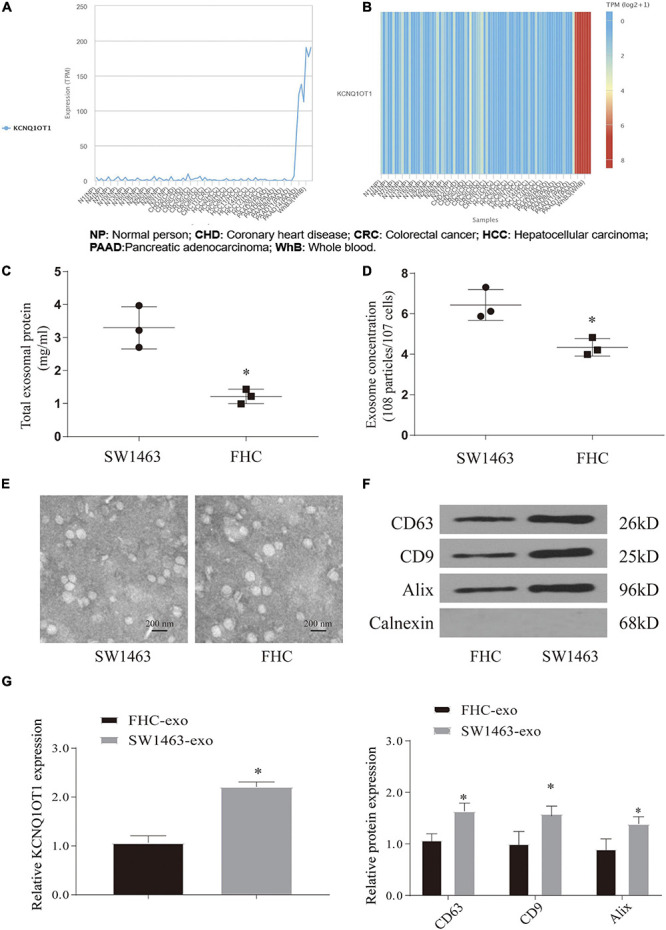
Colorectal cancer (CRC) cells can release a large amount of exosomes containing KCNQ1OT1. **(A,B)** Analysis of the expression difference of KCNQ1OT1 in different tumor cell exosomes in exoRbase database. **(C)** Total exosome protein expression. **(D)** The concentration of exosomes secreted in different cells by nanoparticle tracking analysis (NTA). **(E)** Morphology of exosomes in each group under electron microscope. **(F)** Expression of exosome markers in different tumor cells. **(G)** The expression of KCNQ1OT1 in exosomes derived from different cells. ^∗^*p* < 0.05 compared with the FHC-exo group.

Through the above research, we can see that the expression of KCNQ1OT1 in CRC tissues is significantly increased and combined with our previous research results. Therefore, the source of the increase has attracted the attention of this research group. Exosomes are carriers that can carry and release small molecules well. Therefore, referring to the aforementioned method (Li S. et al., 2018), we tried to analyze whether CRC tissue exosomes carry KCNQ1OT1 through the exoRbase database^3^. The predicted results are exciting. It showed that the expression of KCNQ1OT1 in CRC-derived exosomes was significantly increased, which provided us with ideas for further research (Figures 2A,B). Our further analysis of SW1463 secreting exosomes showed that the number of exosomes secreted by tumor cells increased compared with that in normal cells (*p* < 0.05) (Figures 2C,D,F). However, the size and shape of exosomes from different sources did not change (Figure 2E). It was found that KCNQ1OT1 was significantly increased in the exosomes secreted by SW1463 by detecting the contents of exosomes (*p* < 0.05) (Figure 2G). These results show that CRC cells can increase the expression of KCNQ1OT1 through the number and content of exosomes secreted. Therefore, we will verify its action form and status in the next step.

### Exosomes Promote the Proliferation and Invasion of Colorectal Cancer

**FIGURE 3:**
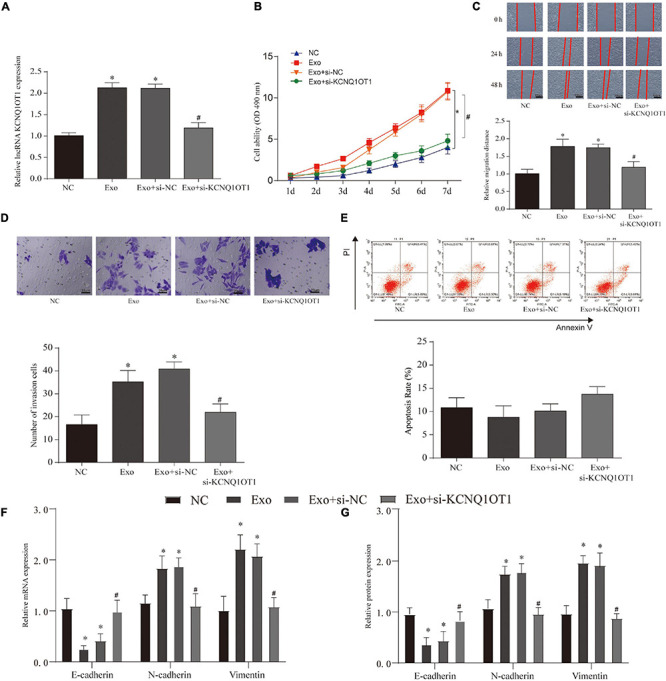
Colorectal cancer (CRC)-derived exosomes promote the proliferation and invasion of CRC in the *in vitro* assay. **(A)** KCNQ1OT1 expression in different treatment groups. **(B)** Cell Counting Kit (CCK)-8 is used to analyze the proliferation ability of CRC cells after different treatments. **(C,D)** Wound healing and Transwell assay were used to detect the migration and invasion ability of CRC cells after different treatments. **(E)** Detection of CRC cell apoptosis in different treatment groups. **(F,G)** Expression of epithelial–mesenchymal transition (EMT)-related genes and proteins. ^∗^*p* < 0.05 compared with the NC group, *^#^p* < 0.05 compared with the Exo+si-NC group.

In order to verify the effect of exosomes on tumor cells, we constructed a coculture system of these. According to the process of PKH26 labeling exosomes into cells, the exosomes successfully entered SW1463 cells and increased with time (Supplementary Figure 1B). In addition, we used CCK-8 assay, wound healing assay, and Transwell assay to test the effect of exosomes on tumor cells and verify whether they play a role through KCNQ1OT1. The results showed that coculture of exosomes can significantly increase the expression of KCNQ1OT1 (*p* < 0.05) (Figure 3A). The tumor cell proliferation, migration, and invasion ability was significantly enhanced compared with the NC group. Only when KCNQ1OT1 was inhibited, this cancer-promoting effect was reversed (*p* < 0.05) (Figures 3B–D). When we tested the apoptosis of tumor cells, we found that although the coculture of exosomes and SW1463 can reduce tumor cell apoptosis to a certain extent, the difference is not statistically significant (Figure 3E). In addition, we found the same phenomenon after testing the markers in the epithelial–mesenchymal transition (EMT) process (Figures 3F,G).

**FIGURE 4:**
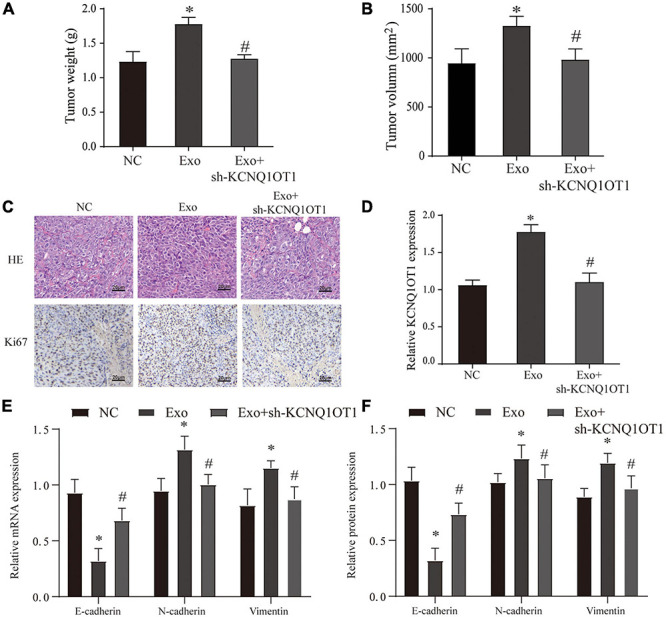
Colorectal cancer (CRC)-derived exosomes promote the proliferation and invasion of CRC in the *in vivo* assay. **(A,B)** Tumor size and weight after different treatments in BALB/c nude mice. **(C)** H&E and Ki67 immunohistochemical staining were used to detect tumor proliferation in different groups. **(D)** The expression of KCNQ1OT1 in tumor tissues from different treatment groups. **(E,F)** Expression of epithelial–mesenchymal transition (EMT)-related genes and proteins. ^∗^*p* < 0.05 compared with the NC group, ^#^*p* < 0.05 compared with the Exo group, *N* = 6.

In the *in vivo* assays, we found that the size, weight, EMT, and proliferation of tumors have been significantly improved after exosome treatment. This phenomenon disappears only when KCNQ1OT1 is suppressed (*p* < 0.05) (Figure 4).

### LncRNA KCNQ1OT1 Can Promote Immune Escape of Colorectal Cancer Cells

**FIGURE 5:**
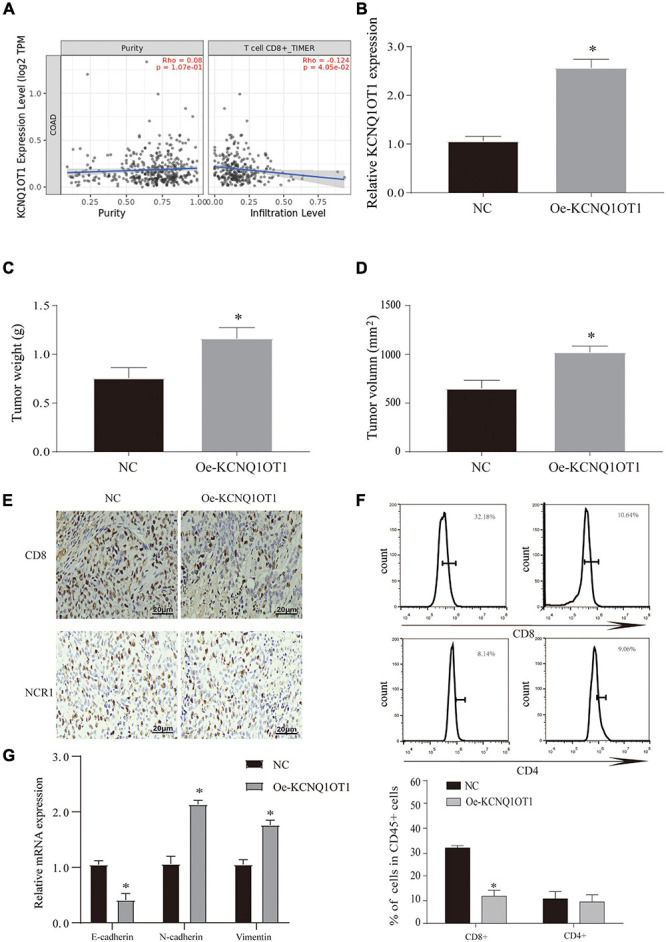
Long non-coding RNA (lncRNA) KCNQ1OT1 can promote immune escape of colorectal cancer (CRC) cells. **(A)** The TIMER 2.0 database is used to detect the correlation between KCNQ1OT1 and CD8+ T cells. **(B)** The expression of KCNQ1OT1 in tumor tissues from different treatment groups in BALB/c mice. **(C,D)** Tumor size and weight after different treatments in BALB/c mice. **(E)** Immunohistochemistry was used to detect the expression of CD8 and NCR1 in tumor tissues. NCR1 was used as a marker of natural killer cells. **(F)** Flow cytometry is used to detect the content of CD8+ and CD4+ T cells in tumor tissues. **(G)** The expression of epithelial–mesenchymal transition (EMT)-related proteins. ^∗^*p* < 0.05 compared with the NC group, *N* = 6.

Although through these results we have verified that KCNQ1OT1 can promote tumor development through the autocrine effect of exosomes, its action form and pathway still bother us. According to other studies, changes in the tumor microenvironment (TME) play an important role in the development of tumors, and the changes in the immune microenvironment have gradually attracted people’s attention (Badawi et al., 2015). We tried to use the TIMER 2.0 database^4^ to try to analyze its possible connection with the immune microenvironment and found that KCNQ1OT1 is negatively correlated with CD8+ T cells in CRC (Figure 5A). Therefore, we constructed a tumor model of KCNQ1OT1 in wild mice, and the results showed that tumor weight and volume were significantly increased when KCNQOT1 expression was elevated (*p* < 0.05) (Figures 5B–D). After staining CD8 and NCR1, it was found that overexpression of KCNQ1OT1 can affect cytotoxic T cells to cause immune escape. This phenomenon has nothing to do with natural killer cells and CD4+ T cells (*p* < 0.05) (Figures 5E,F). Finally, we also detected the expression of EMT-related genes and found that KCNQ1OT1 may lead to the enhancement of metastasis and invasion ability through such immune escape phenomenon (Figure 5G).

**FIGURE 6:**
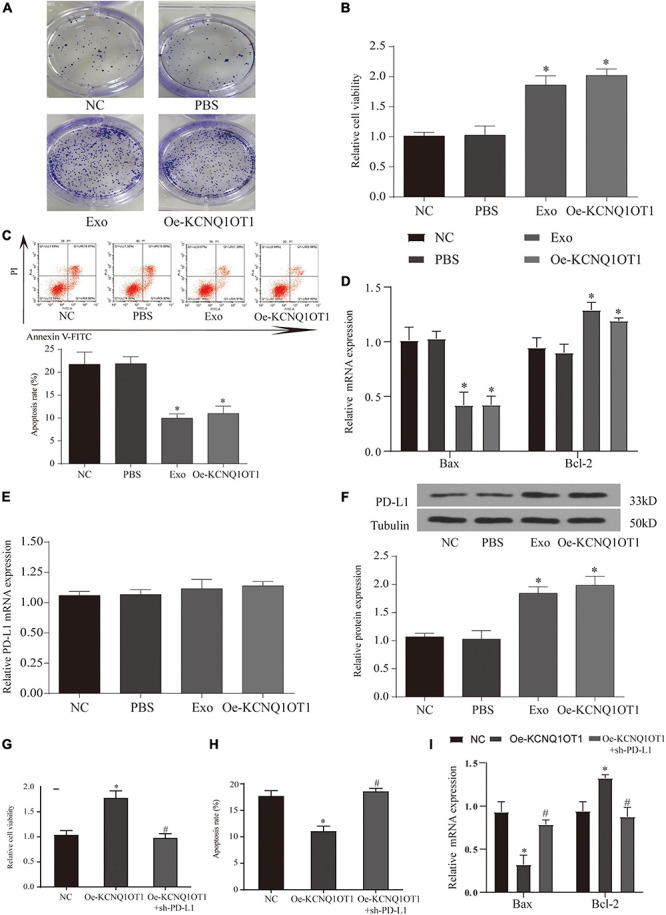
Long non-coding RNA (lncRNA) KCNQ1OT1 regulates tumor immune escape through programmed death-ligand 1 (PD-L1). **(A)** Activated T cell and SW1463 cells were cocultured in six-well plates for 7 days, and then surviving SW1463 cells were visualized by colony formation assay. **(B)** T cell-mediated SW1463 cell killing analysis measured by Cell Counting Kit (CCK)-8 assay. **(C)** Cell-mediated SW1463 cell killing analysis measured by apoptosis detection. **(D)** Cell-mediated SW1463 cell killing analysis measured by apoptosis detection. **(E,F)** PD-L1 mRNA and protein expression of SW1463 cells in different treatment groups. **(G)** T cell-mediated SW1463 cell killing analysis measured by CCK-8 assay. **(H,I)** Cell-mediated SW1463 cell killing analysis measured by apoptosis detection. ^∗^*p* < 0.05 compared with the NC/PBS group, ^#^*p* < 0.05 compared with the Oe-KCNQ1OT1 group.

To confirm the role of KCNQ1OT1 in the immune escape of CRC cells, SW1463 cells were cocultured with activated T cells. We verified the expression of KCNQ1OT1 in each treatment group (Supplementary Figure 2A). Overexpression of KCNQ1OT1 significantly inhibited T cell-mediated cell killing (*p* < 0.05) (Figures 6A–D).

### LncRNA KCNQ1OT1 Regulates Tumor Immune Escape Through Programmed Death-Ligand 1

The combination of PD-L1 on tumor cells and PD-1 on T cells causes T cells to be suppressed, thereby bypassing immune surveillance (Calderaro et al., 2016). Therefore, we want to explore whether the dysregulation of PD-L1 in cancer cells is involved in KCNQ1OT1-mediated tumor growth. We tested the expression of PD-L1 in each treatment group cocultured with T cells, and the results showed that the protein expression of PD-L1 in the treatment group with high KCNQ1OT1 expression increased significantly (*p* < 0.05) (Figure 6F), but the mRNA did not change significantly (*p* > 0.05) (Figure 6E). In our second T-cell killing experiment, we interfered with the expression of PD-L1, trying to clarify the regulatory relationship between KCNQ1OT1 and PD-L1. The results showed that when PD-L1 expression was inhibited, the immune escape phenomenon caused by KCNQ1OT1 was reversed. These results confirm that PD-L1 participates in the phenomenon of KCNQ1OT1-mediated tumor immune escape (*p* < 0.05) (Figures 6G–I).

### Ubiquitination Is Involved in the Immune Escape Phenomenon Regulated by Programmed Death-Ligand 1

**FIGURE 7:**
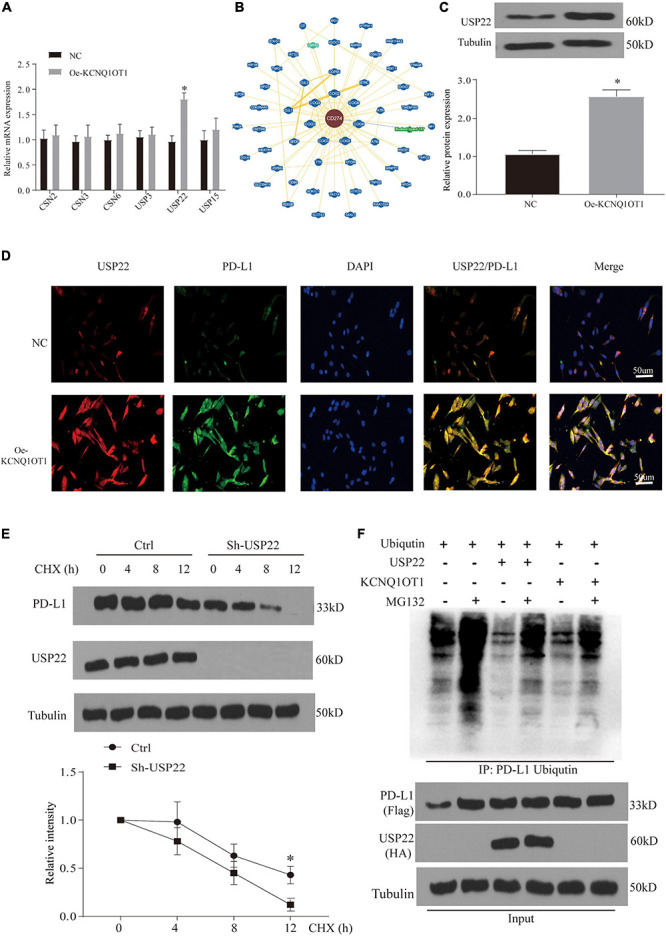
Ubiquitination is involved in the immune escape phenomenon regulated by programmed death-ligand 1 (PD-L1). **(A)** Quantitative reverse transcription PCR (RT-qPCR) analysis of the deubiquitinating enzymes in SW1463 cells; glyceraldehyde 3-phosphate dehydrogenase (GAPDH) is used as an internal reference gene. **(B)** Interaction network of PD-L1 analyzed in BioGRID database. **(C)** Differential expression of ubiquitin-specific peptidase 22 (USP22) protein in different treatment groups of tumor tissues. **(D)** Immunofluorescence staining of USP22 (red) and PD-L1 (green) in SW1463 cells. Scale bar, 30 μm. **(E)** PD-L1 protein half-life determination in SW1463 cells lacking USP2. Before Western blot analysis, cells were co-incubated with cycloheximide (CHX, 50 μg/ml) at the specified time points. **(F)** After treatment with 10 μM MG-132 for 3 h or 8 h, the ubiquitination of PD-L1 in HEK293 cells was determined. Cells were transfected with ubiquitin after different treatments. The ubiquitinated PD-L1 was subjected to immunoprecipitation (IP) before Western blot analysis with ubiquitin antibody. ^∗^*p* < 0.05 compared with the NC group.

In response to the above results, we further verified the increase in protein in patient samples and TCGA data. The results also showed that the expression of PD-L1 mRNA in tumor patients remained unchanged, but the protein increased significantly (Supplementary Figures 2B–E). These phenomena indicate that KCNQ1OT1 may affect the posttranslational modification of PD-L1. Ubiquitination is the most studied posttranslational modification. In the process of ubiquitination, Ub binds to the target protein to regulate its degradation (Sun et al., 2015). By consulting the literature, we screened several genes from different deubiquitination families for verification. RT-PCR results showed that the difference in the increase of USP22 was the most significant after overexpression of KCNQ1OT1 (*p* < 0.05) (Figure 7A). In addition, we found that USP22 has stronger evidence in the protein that interacts with PD-L1 in the BioGRID data verification (Figure 7B). Similar results were also verified at the protein expression level (*p* < 0.05) (Figure 7C). This result was confirmed by immunofluorescence staining, which showed that USP22 and PD-L1 were co-localized in SW1463 cells (Figure 7D). In addition, through half-life determination, we found that PD-L1 is rapidly degraded in cells lacking USP22 (*p* < 0.05) (Figure 7E). Next, we used the proteasome inhibitor MG-132 to evaluate the deubiquitin effect of USP22 on PD-L1. Although MG-132 significantly enhanced the accumulation of Ub-PD-L1, USP22 overexpression or KCNQ1OT1 overexpression treatment significantly reduced its accumulation (Figure 7F).

### MiR-30a-5p Is Involved in the Regulation of LncRNA KCNQ1OT1 on USP22

**FIGURE 8:**
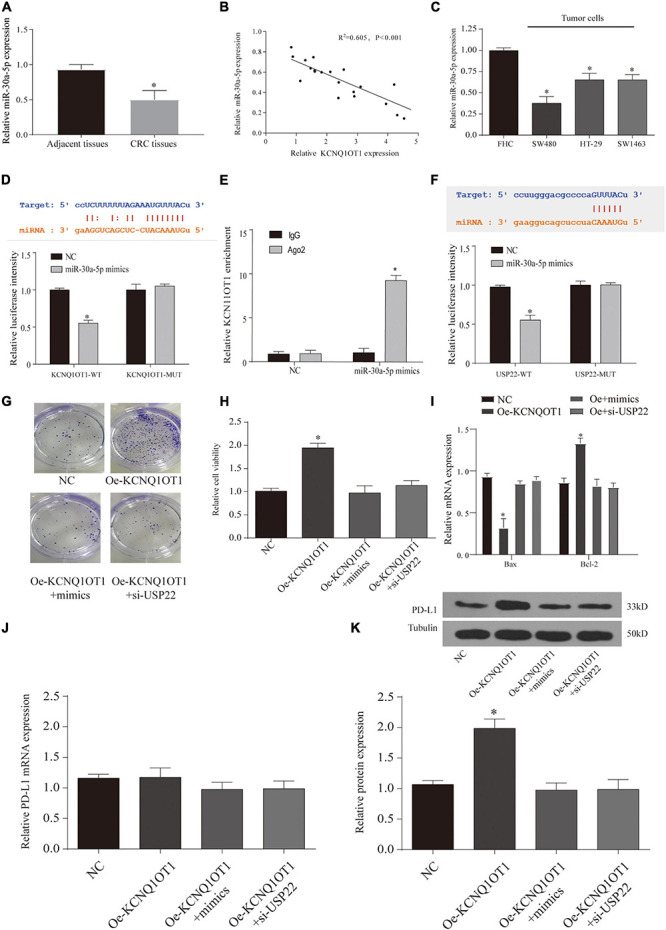
Long non-coding RNA (lncRNA) KCNQ1OT1 regulates tumor cell immune escape *via* miR-30a-5p/ubiquitin-specific peptidase 22 (USP22) in the *in vitro* assay. **(A)** The expression of miR-30a-5p in patient samples. **(B)** The correlation between miR-30a-5p and lncRNA KCNQ1OT1 in tumor tissues. **(C)** Expression of miR-30a-5p in different types of colorectal cancer (CRC) cells. **(D,E)**. Dual-luciferase report and ribonucleoprotein immunoprecipitation (RIP) assay verify the targeting of lncRNA KCNQ1OT1 and miR-30a-5p. The binding site prediction comes from the Starbase database. **(F)** Dual-luciferase report verifies the targeting of USP22 and miR-30a-5p. The binding site prediction comes from the Starbase database. **(G)** Activated T cell and SW1463 cells were cocultured in six-well plates for 7 days, and then surviving SW1463 cells were visualized by colony formation assay. **(H,I)** T cell-mediated SW1463 cell killing analysis measured by Cell Counting Kit (CCK)-8 assay and detection of apoptotic protein. **(J,K)** Programmed death-ligand 1 (PD-L1) protein expression of SW1463 cells in different treatment groups. ^∗^*p* < 0.05 compared with the relative control group.

Through the results of Figure 7A, we found that lncRNA KCNQ1OT1 and USP22 have the same trend of action, so we tried to find the intermediate link of its regulation. First, we screened the difference of miRNA between the tumor tissue treated with exosomes and the NC group through microarray analysis (Supplementary Figure 1C). In addition, we used the data of Starbase, miRDB, and lncBase to screen the target genes and combined with the microarray results. We selected three miRNAs (miR-330-3p, miR-30a-5p, and miR-515-5p) for expression verification (Supplementary Figure 1D). As a result, only miR-30a-5p had the most significant difference, and after verification by TCGA database, it was found that miR-30a-5p and KCNQ1OT1 had a negative correlation in tumor patients (Supplementary Figures 1E,F). In addition, we also verified the low expression of miR-30a-5p in tumor tissues and adjacent tissues (*p* < 0.05) (Figures 8A,B). This phenomenon has also been confirmed in cell experiments (*p* < 0.05) (Figure 8C). Then, we used the Starbase database to predict their possible binding sites, and through the dual-luciferase reporter assay and RIP assay, we proved that the three are mutually binding (*p* < 0.05) (Figures 8D–F). Furthermore, after verifying the downstream target genes by overexpression of KCNQ1OT1 and miR-30a-5p (Supplementary Figures 3A,B), we found that lncRNA can competitively bind miR-30a-5p to affect the expression of USP22.

### LncRNA KCNQ1OT1 Regulates Tumor Cell Immune Escape *via* MiR-30a-5p/USP22

**FIGURE 9:**
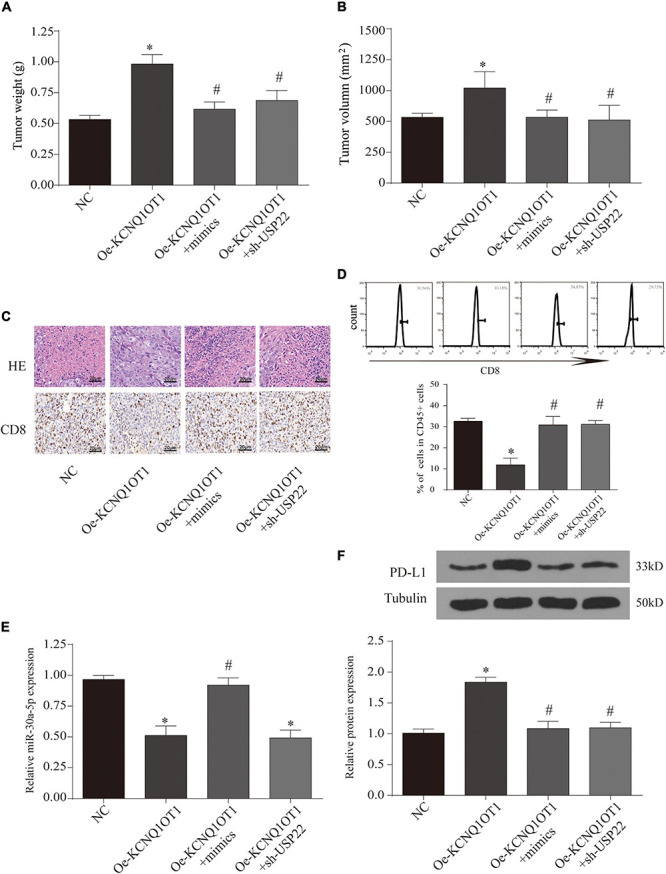
Long non-coding RNA (lncRNA) KCNQ1OT1 regulates tumor cell immune escape *via* miR-30a-5p/ubiquitin-specific peptidase 22 (USP22) in the *in vivo* assay. **(A,B)** Tumor size and weight after different treatments in BALB/c mice. **(C)** Hematoxylin–eosin staining and CD8 immunohistochemical staining were used to detect tumor proliferation and immune escape in different groups. **(D)** Flow cytometry is used to detect the content of CD8+ T cells in tumor tissues. **(E)** The expression of miR-30a-5p in tumor tissues. **(F)** Programmed death-ligand 1 (PD-L1) protein expression of tumor tissues in different treatment groups. ^∗^*p* < 0.05 compared with the NC group, ^#^*p* < 0.05 compared with the Oe-KCNQ1OT1 group, *N* = 6.

Finally, we observe the relationship between the three through T-cell killing experiments. The results show that overexpression of miR-30a-5p or inhibition of USP22 can reverse the inhibition of T-cell function caused by the high expression of KCNQ1OT1 (*p* < 0.05) (Figures 8G–I). The expression of PD-L1 was also reversed (*p* < 0.05) (Figures 8J,K). Similarly, we further verified this phenomenon through animal models. The results showed that the group that overexpressed miR-30a-5p and suppressed USP22 showed a decrease in tumor size and weight (*p* < 0.05) (Figures 9A,B). The results of immunohistochemistry and CD8 analysis further confirmed that this immune escape phenomenon in tumors has been reversed to a certain extent (*p* < 0.05) (Figures 9C,D). This reversal is achieved by regulating the expression of PD-L1 and miR-30a-5p (*p* < 0.05) (Figures 9E,F).

## Discussion

**FIGURE 10:**
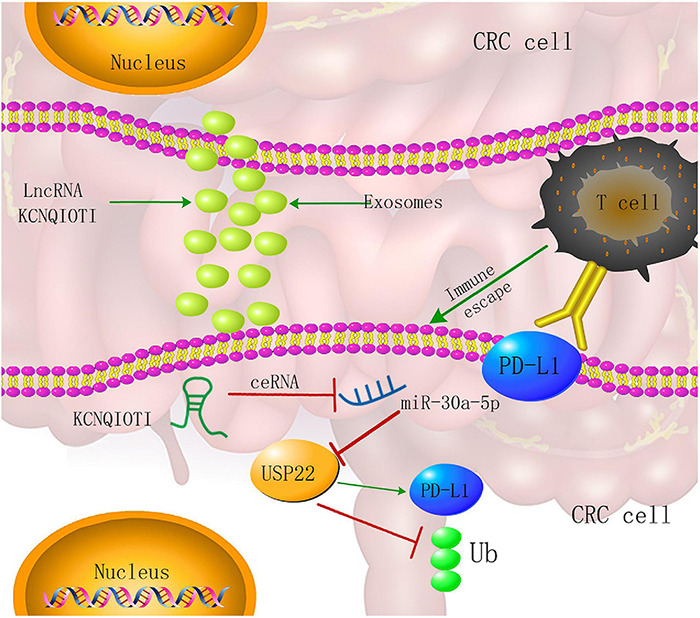
Colorectal cancer (CRC) cell-derived exosome KCNQ1OT1 regulates the mechanism of tumor cell formation through the miR-30a-5p/ubiquitin-specific peptidase 22 (USP22)/programmed death-ligand 1 (PD-L1) axis.

Our findings reveal a novel immune escape mechanism involving exosomes, non-coding RNA, CRC cells, and CD8+ T cells. Among them, the tumor-promoting effect of lncRNA KCNQ1OT1 is through the autocrine effect of tumor cell-derived exosomes, which mediates the miR-30a-5p/USP22 pathway to regulate the ubiquitination of PD-L1 and inhibits CD8+ T-cell response, thereby promoting CRC development (Figure 10). Through these findings, we will provide a reference basis for future tumor immunotherapy and targeted therapy.

LncRNA was originally thought to be a by-product of RNA polymerase II transcription, a kind of “noise” and no biological function. However, recent studies have shown that lncRNA can regulate gene expression on multiple levels (epigenetic regulation, transcription regulation, posttranscriptional regulation, etc.) and participate in the pathological process of many diseases, especially in malignant tumors (Lin and Yang, 2018). In addition, lncRNAs also act as competing endogenous RNA (ceRNA), interacting with miRNAs, and by affecting the expression of messenger RNA (mRNA) (Li S. et al., 2020). Zhu et al. (2020) found a new type of oncogenic lncRNA RP11-757G1.5 through microarray analysis, which is overexpressed in CRC tissues, especially in aggressive cases. In addition, the upregulation of RP11-757G1.5 is closely related to the adverse clinical outcome of CRC patients. Further functional analysis showed that RP11-757G1.5 can promote cell proliferation *in vitro* and *in vivo*. The mechanism for this phenomenon is that RP11-757G1.5 regulates the expression of YAP1 and promotes the development of CRC by competitively binding miR-139-5p to inhibit its activity (Zhu et al., 2020). In this study, we combined the previous research results to screen and verify the high expression of lncRNA KCNQ1OT1. Through the ROC curve, we found that it has a certain diagnostic value. In further research, we tried to explore the source of KCNQ1OT1. Therefore, the consideration of exosomes appeared in this study, and our conjecture was confirmed to a certain extent by means of bioinformatics.

Our results show that CRC cells can autocrinely regulate their own processes by increasing the secretion of exosomes. In recent years, due to its ability to carry target molecules/proteins and target related cells, its research has gradually attracted people’s attention. Due to its own characteristics, the gradual deepening of its mechanism can provide a good carrier and interference possibility for future targeted therapy (Han et al., 2020). Similar studies have been reported on CRC, which are similar to the ideas and conclusions of this study (Liang et al., 2019). However, a large number of them are relatively superficially studied, and the feasibility of combining with clinical treatment is poor. Therefore, we need to continuously explore the downstream mechanisms.

TME is composed of a variety of cells, extracellular matrix, growth factors, and proteolytic enzymes and their inhibitors, signal molecules (Junttila and de Sauvage, 2013). TME mainly alters tumor growth, metastasis, and prognosis by affecting malignant cells. The origin, development, invasion, and metastasis of the TME are all regulated by the extracellular matrix, cytokines, and immune cells in the microenvironment. In turn, TME can change the proliferation, metastasis and prognosis of tumors by affecting the cellular immune microenvironment (Shen et al., 2020). Tumor cells can escape immune surveillance and induce immune tolerance in a variety of ways, including the release of exosomes (Whiteside, 2016). A number of studies have focused on the complex crosstalk between TME and CD8+ T cells (Cai et al., 2019). Interestingly, we found that KCNQ1OT1 can affect CD8+ T cells through the bioinformatics database. Through our research, we found that there is no statistical difference in the effects of KCNQ1OT1 on natural killer cells and CD4+ T cells. This part of the results is similar to a previous study (Lou et al., 2019). However, the apoptosis detection of SW1463 cocultured with T cells found that although overexpression of KCNQ1OT1 reduced tumor cell apoptosis to a certain extent, there was no statistical difference in this apoptosis. This may be caused by the low basic apoptosis rate of tumor cells. The main manifestations of cancer promotion may be proliferation and invasion.

Numerous studies have shown that PD-1 is the main and key inhibitory receptor in tumor immunity. Immunotherapy aimed at blocking PD-1 and its ligand (PD-L1) has become an important means of treating tumors (Chen Z. et al., 2020). It is precisely because we did not find significant differences in the detection of PD-L1 mRNA expression. Therefore, the role of ubiquitination here has attracted our attention.

Our results prove the role of deubiquitinating enzyme USP22 in this respect. In addition, through the screening of miRNAs, we found that miR-30a-5p may serve as an intermediate counterattack in the regulation of KCNQ1OT1 and USP22. At present, a large number of studies have discovered and verified the role of miR-30a-5p in CRC (Sun et al., 2019; Li J. et al., 2020). However, its role as an intermediate pathway between KCNQ1OT1 and USP22 to regulate tumor immune escape is the first report and verification in this study.

## Conclusion

We found that the role of exosomes in PD-L1 regulation through the KCNQ1OT1/miR-30a-5p/USP22 axis provides a way to explore the phenomenon of CRC immune escape. At the same time, the adjuvant therapy and targeted therapy for the clinical application of PD-L1 inhibitors provide new ideas. However, despite the extensive verification of this approach *in vivo* and *in vitro*, this study still lacks corresponding clinical data as support. Therefore, in the future, we still need to further carry out clinical sample detection and intervention to further strengthen the treatment of CRC and improve the survival rate of patients.

## Data Availability Statement

The original data presented in the study are included in the article/Supplementary Material. Further inquiries can be directed to the corresponding author. And the microarray data can also be found here https://www.frontiersin.org/articles/10.3389/fcell.2021.653808/full#supplementary-material.

## Ethics Statement

The animal study was reviewed and approved by the Ethical Committee of Sichuan Provincial People’s Hospital, University of Electronic Science and Technology of China.

## Author Contributions

DX, LN, and JZ gave substantial contributions to the conception and the design of the manuscript. DX and LW contributed to the acquisition, analysis, and interpretation of the data. LN and JZ revised the manuscript critically. All authors have participated to drafting the manuscript, read and approved the final version of the manuscript.

## Conflict of Interest

The authors declare that the research was conducted in the absence of any commercial or financial relationships that could be construed as a potential conflict of interest.

## Footnotes

1 https://www.r-project.org/

2 http://starbase.sysu.edu.cn/

3 http://www.exorbase.org/

4 http://timer.comp-genomics.org/

## Abbreviations

CRC: colorectal cancer
LncRNA: long non-coding RNA
Exo: exosomes
DUBs: ubiquitinating enzymes
Ub: ubiquitin
USP22: ubiquitin-specific peptidase 22
ATCC: American Type Culture Collection
RNA-Seq: RNA sequencing
NTA: nanoparticle tracking analysis
RIP: ribonucleoprotein immunoprecipitation
RT-qPCR: quantitative reverse transcription PCR
IHC: immunohistochemistry
WB: Western blot
ceRNA: competing endogenous RNA
mRNA: messenger RNA
TME: tumor microenvironment
PD-1: programmed death receptor 1.
