# Supplementary material for: LncRNA KCNQ1OT1 Secreted by Tumor Cell-Derived Exosomes Mediates Immune Escape in Colorectal Cancer by Regulating PD-L1 Ubiquitination via MiR-30a-5p/USP22
Source: Front Cell Dev Biol. 2021 Jul 19;9:653808. doi: 10.3389/fcell.2021.653808 (PMC8326752; doi:10.3389/fcell.2021.653808)

Supplementary Table 1. Base line data of the patients

Characteristics		The expression of KCNQ1OT1		
		low expression(n=10)	high expression(n=10)	*P* value
Gender	Male	5	6	1.000
	Female	5	4	
Age(years)	<60	4	4	1.000
	≥60	6	6	
Lymph node metastasis	Absence	3	1	**0.022***
	Presence	7	9	

* *P*<0.05 by Chi-Square test

Supplementary Table 2. patients information of cases used for lncRNA analysis

Case number	Age	Gender	T stage	N stage	M stage	Stage	colon polyps present	lymph node examined count	lymphatic invasion
1	68	Female	T1	N0	M0	Stage I	Not Available	Not Available	NO
2	48	Female	T1	N0	M0	Stage II	NO	17	NO
3	65	Male	T3	N1	M1	Stage IV	Not Available	34	Not Available
4	73	Female	T2	N0	M0	Stage I	NO	24	NO
5	54	Male	T3	N0	M0	Stage IIA	YES	32	NO
6	50	Male	T3	N2b	M1	Stage IIIC	NO	15	NO
7	54	Female	T3	N0	M0	Stage IIA	YES	12	NO
8	73	Female	T3	N2	M0	Stage II	Not Available	17	YES

Supplementary table 3. Primer sequence

	**Primer Sequence**
miR-30a-5p forward	5'- GGCGTGTAAACATCCTCGACTG -3 '
miR-30a-5p reverse	5'- GTGCAGGGTCCG AGGT -3'
U6 forward	5’-CTCGCTTCGGCAGCACATA-3’
U6 reverse	5’-AACGATTCACGAATTTGCGT-3’
USP22 forward	5’-CCCCTCACATCCCGTATA-3’
USP22 reverse	5’-TTCCCATTGTCATCACCTT-3’
PD-L1 forward	5’-GCCGACTACAAGCGAATTAC-3’
PD-L1 reverse	5’-TCTCAGTGTGCTGGTCACAT-3’
KCNQ1OT1 forward	5’-TGCAGAAGACAGGACACTGG-3’
KCNQ1OT1 reverse	5’-CTTTGGTGGGAAAGGACAGA-3’
GAPDH forward	5’-GAAGGTGAAGGTCGGAGTC-3’
GAPDH reverse	5’-GAAGATGGTGATGGGATTTC-3’

Supplementary Figure 1 Differential gene screening of different samples

(A). The heat-map of top10 upregulated and down-regulated lncRNAs in CRC samples in comparison with corresponding tissues. (B). Immunofluorescence staining uses PKH26 to label exosomes to detect the entry of exosomes into SW1463 at 2h and 24h, respectively. (C). Heat map is used to show the differential expression of miRNA between tumor tissues treated with exosomes and untreated tumor tissues. (D). Venn diagram for screening miRNA. (E). The differential expression of miR-330-3p, miR-30a-5p and miR-515-5p in tumor tissues after overexpression of KCNQ1OT1 was detected. (F). TCGA database is used to analyze the correlation between KCNQ1OT1 and miR-30a-5p in tumor tissues.

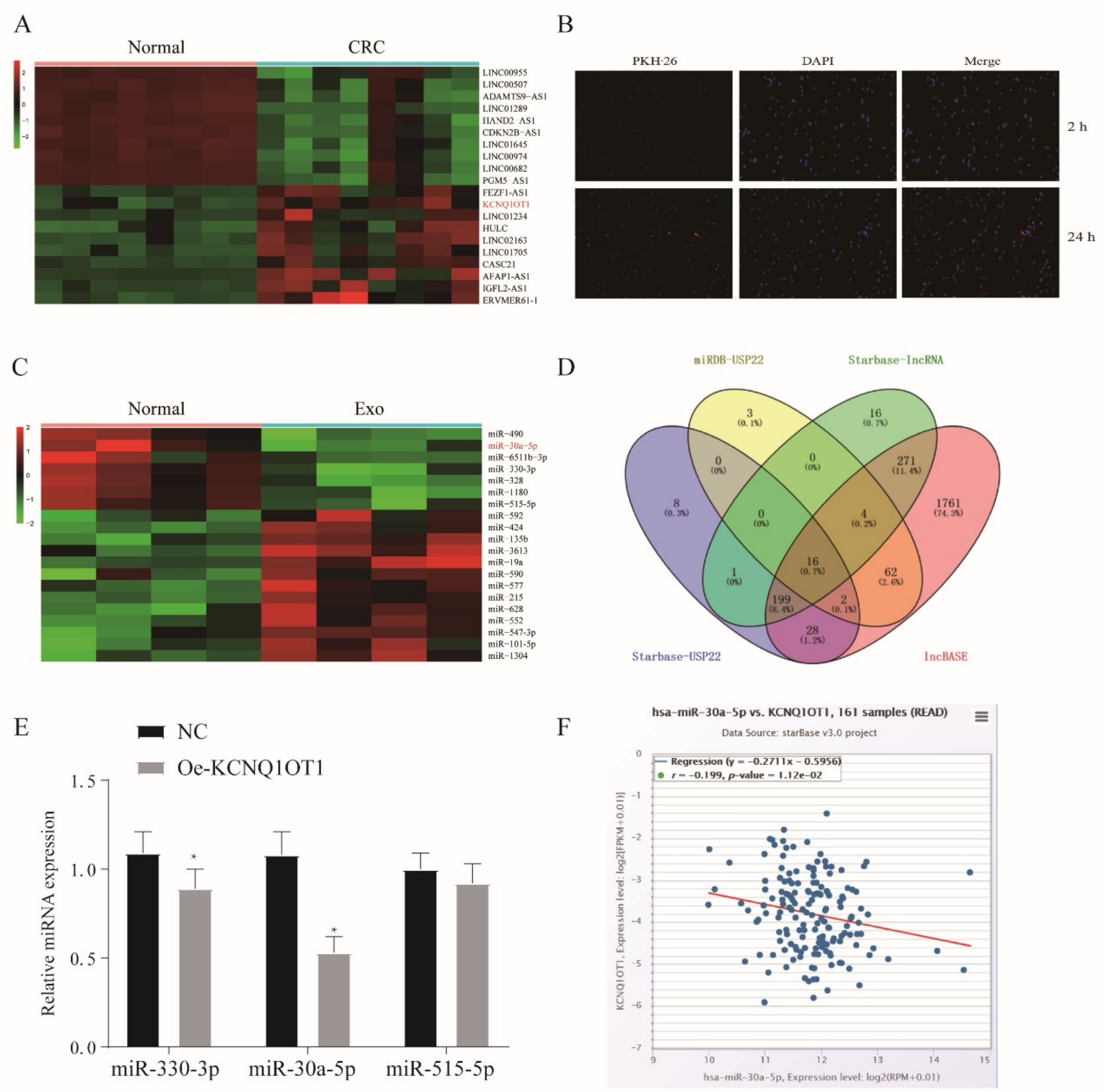

Supplementary Figure 2 Detection of PD-L1 and KCNQ1OT1 expression in different tissues

(A). The expression of KCNQ1OT1 in tumor tissues from different treatment groups. (B, D).PD-L1mRNA and protein expression in patients tisusses. (C). TCGA database verifies the expression of PD-L1mRNA in tumor tissues. (E). Expression of PD-L1mRNA in different types of CRC cells. (F). PD-L1 protein expression in different groups. *P<0.05, compared with relative control group.

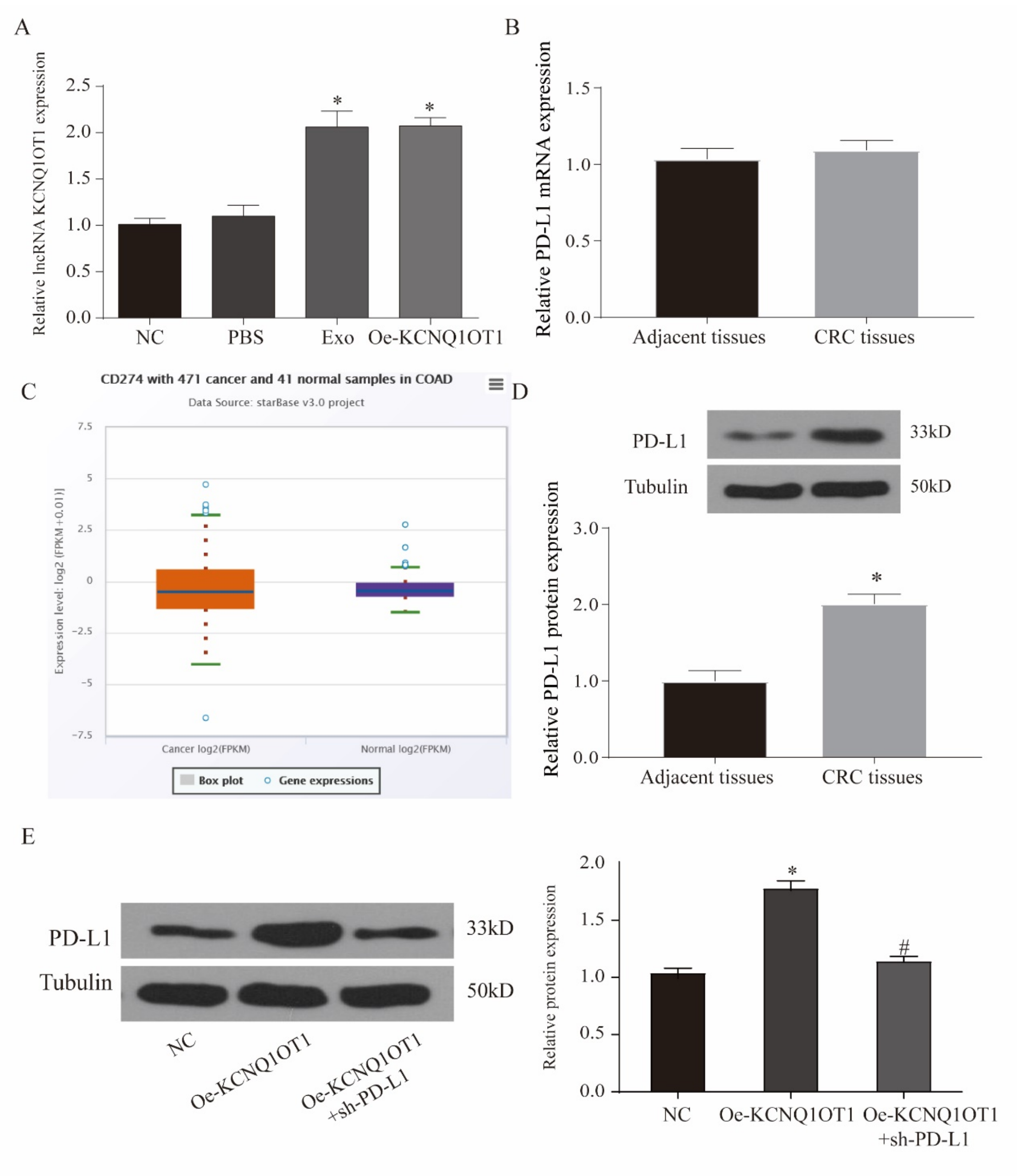

Supplementary Figure 3 KCNQ1OT1, miR-30a-5p and USP22 regulatory relationship detection.

(A). RT-qPCR is used to detect the expression of miR-30a-5p after overexpression of KCNQ1OT1. (B). RT-qPCR is used to detect the expression of USP22 after overexpression of miR-30a-5p. **P*<0.05, compared with relative control group.

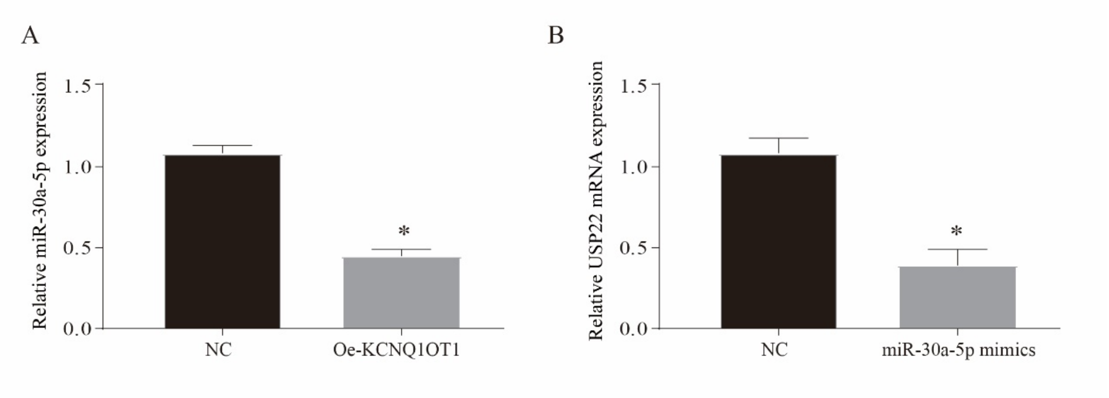

Supplement: Supplementary file 1 [file Presentation_1.pdf]
